# Symmetry reduction and exact solutions of two higher-dimensional nonlinear evolution equations

**DOI:** 10.1186/s13660-017-1587-5

**Published:** 2017-12-21

**Authors:** Yongyi Gu, Jianming Qi

**Affiliations:** 1 School of Mathematics and Information Science, Guangzhou University, Guangzhou, 510006 China; 2 Department of Mathematics and Physics, Shanghai Dianji University, Shanghai, 201306 China

**Keywords:** 30D35, 34A05, nonlinear evolution equations, symmetry, $\exp(-\phi(z))$-expansion method, complex method, exact solutions, meromorphic function

## Abstract

In this paper, symmetries and symmetry reduction of two higher-dimensional nonlinear evolution equations (NLEEs) are obtained by Lie group method. These NLEEs play an important role in nonlinear sciences. We derive exact solutions to these NLEEs via the $\exp(-\phi (z))$-expansion method and complex method. Five types of explicit function solutions are constructed, which are rational, exponential, trigonometric, hyperbolic and elliptic function solutions of the variables in the considered equations.

## Introduction

In 1998, Yu *et al.* [1] extended the Bogoyavlenskii Schiff equation

1 $$ u_{t}+\varPhi (u)u_{s}=0,\quad \varPhi (u)=\partial^{2}_{x}+4u+2u_{x} \partial^{-1}_{x}, $$

to the $(3+1)$-dimensional NLEE

2 $$ \bigl(-4u_{t}+\varPhi (u)u_{s}\bigr)_{x}+3u_{yy}=0, \quad \varPhi (u)=\partial^{2}_{x}+4u+2u_{x}\partial ^{-1}_{x}. $$

Setting $u:=u_{x}$, equation (2) is changed into the $(3+1)$-dimensional potential Yu-Toda-Sasa-Fukuyama (YTSF) equation

3 $$ u_{xxxs}-4u_{xt}+4u_{x}u_{xs}+2u_{xx}u_{s}+3u_{yy}=0. $$

The generalized $(3+1)$-dimensional Zakharov-Kuznetsov (gZK) equation is given by

4 $$ a_{1}u^{2}u_{x}+a_{2}u_{xxx}+a_{3}u_{xyy}+a_{4}u_{xss}+a_{5}u u_{x}+a_{6}u_{xxt}+u_{t}=0. $$

Here $a_{i}$ ($i=1,2,\ldots,6$) are arbitrary constants.

We note that equation (4) includes many famous NLEEs as its special cases. For instance, if $a_{1}=a_{3}=a_{4}=a_{6}=0$, then equation (4) is the Korteweg-de Vries equation [2, 3]. If $a_{2}=a_{4}=a_{5}=0$, then equation (4) is the $(2+1)$ dimensional ZK-MEW equation [4]. If $a_{3}=a_{4}=a_{6}=0$, then equation (4) is the Gardner equation [5]. If $a_{4}=a_{5}=a_{6}=0$, then equation (4) is the modified Zakharov-Kuznetsov equation [6].

In recent years, it has aroused widespread interest in the study of NLEEs [7–13]. Equations (3) and (4) are very meaningful higher-dimensional NLEEs which can describe many dynamic processes and important phenomena in engineering and physics. The YTSF equation is a mostly used model for investigating the dynamics of solitons and nonlinear waves in fluid dynamics, plasma physics and weakly dispersive media [13]. Zakharov and Kuznetsov [14] proposed the ZK equation to describe nonlinear ion-acoustic waves in a plasma comprised of cold ions and hot isothermal electrons in the presence of a uniform magnetic field. Many physical phenomena, in the purely dispersive limit, are governed by this type of equation, such as the long waves on a thin liquid film [15], the Rossby waves in a rotating atmosphere [16], and the isolated vortex of drift waves in a three-dimensional plasma [17]. The gZK equation is of a generalized setting of ZK equation. Seeking exact solutions of NLEEs is an interesting and significant subject. Over the past few years, many powerful methods for constructing the solutions of NLEEs have been used, for instance, the Bäcklund transform method [18], direct algebraic method [19], modified simple equation method [20], Lie group method [21, 22], $\exp(-\phi(z))$-expansion method [8, 9, 23, 24], and so on. Recently, Yuan *et al.* [25–27] introduced the complex method to find the exact solutions of NLEEs in mathematical physics. In this paper, we study symmetries, symmetry reduction of the two higher-dimensional NLEEs, and then we obtain their exact solutions via the $\exp(-\phi(z))$-expansion method and complex method.

## Description of the methods

### Description of the $\exp(-\phi(z))$-expansion method

Suppose that a nonlinear partial differential equation (PDE) is given by

5 $$ P(u,u_{x},u_{y},u_{t},u_{xx},u_{yy},u_{tt}, \ldots)=0, $$

where *P* is a polynomial of an unknown function $u(x,y,t)$ and its derivatives in which nonlinear terms and highest order derivatives are involved. The main steps of this method are given in the following.

Step 1. Substituting the traveling wave transform

$$u(x,y,t)=w(z), \quad z=kx+ly+rt $$

into equation (5) converts it to the following ordinary differential equation (ODE):

6 $$ F\bigl(w,w',w'',w''', \ldots\bigr)=0, $$

in which *F* is a polynomial of $w(z)$ and its derivatives, while $':=\frac{d}{dz}$.

Step 2. Assume that equation (6) has the following traveling wave solution:

7 $$ w(z)=\sum_{j=0}^{n}C_{j}\bigl( \exp\bigl(-\phi(z)\bigr)\bigr)^{j}, $$

where $C_{j}$ ($0\leq j\leq n$) are constants to be determined, such that $C_{j}\neq0$ and $\phi=\phi(z)$ satisfies the ODE as follows:

8 $$ \phi'(z)=\exp\bigl(-\phi(z)\bigr)+\mu\exp\bigl(\phi(z)\bigr)+ \delta. $$

Equation (8) has the following solutions.

When $\delta^{2}-4\mu>0$, $\mu\neq0$,

9 $$\begin{aligned}& \phi(z)=\ln\biggl(\frac{-\sqrt{(\delta^{2}-4\mu)}\tanh(\frac{\sqrt{\delta^{2}-4\mu }}{2}(z+c)-\delta)}{2\mu}\biggr), \end{aligned}$$

10 $$\begin{aligned}& \phi(z)=\ln\biggl(\frac{-\sqrt{(\delta^{2}-4\mu)}\coth(\frac{\sqrt{\delta^{2}-4\mu }}{2}(z+c)-\delta)}{2\mu}\biggr). \end{aligned}$$

When $\delta^{2}-4\mu<0$, $\mu\neq0$,

11 $$\begin{aligned}& \phi(z)=\ln\biggl(\frac{\sqrt{(4\mu-\delta^{2})}\tan(\frac{\sqrt{(4\mu-\delta ^{2})}}{2}(z+c)-\delta)}{2\mu}\biggr), \end{aligned}$$

12 $$\begin{aligned}& \phi(z)=\ln\biggl(\frac{\sqrt{(4\mu-\delta^{2})}\cot(\frac{\sqrt{(4\mu-\delta ^{2})}}{2}(z+c)-\delta)}{2\mu}\biggr). \end{aligned}$$

When $\delta^{2}-4\mu>0$, $\mu=0$, $\delta\neq0$,

13 $$ \phi(z)=-\ln\biggl(\frac{\delta}{\exp(\delta(z+c))-1}\biggr). $$

When $\delta^{2}-4\mu=0$, $\mu\neq0$, $\delta\neq0$,

14 $$ \phi(z)=\ln\biggl(-\frac{2(\delta(z+c)+2)}{\delta^{2}(z+c)}\biggr). $$

When $\delta^{2}-4\mu=0$, $\mu=0$, $\delta=0$,

15 $$ \phi(z)=\ln(z+c). $$

Here $C_{n}\neq0$, *δ*, *μ* are constants that will be determined later and *c* is an arbitrary constant. We take the homogeneous balance between nonlinear terms and highest order derivatives of equation (6) to determine the positive integer *n*.

Step 3. Substituting equation (7) into equation (6) and accounting the function $\exp(-\phi(z))$, we obtain a polynomial of $\exp(-\phi(z))$. Equating all the coefficients of the same power of $\exp(-\phi(z))$ to zero yields a set of algebraic equations. By solving the algebraic equations, we get the values of $C_{n}\neq0$, *δ*, *μ*, and then we substitute them into equation (7) along with equations (9)-(15) to complete the determination of the solutions of equation (5).

### Description of the complex method

Let $m\in{\mathbb {N}}^{*}:=\{1, 2, 3,\ldots\}$, $r_{j}\in{\mathbb {N}}={\mathbb {N}}^{*}\cup\{0\}$, $j=0, 1, \ldots, m$, $r=(r_{0}, r_{1},\ldots, r_{m})$, and

$$K_{r}[w](z):=\prod_{j=0}^{m} \bigl[w^{(j)}(z)\bigr]^{r_{j}}, $$

then $d(r):=\sum_{j=0}^{m}r_{j}$ is the degree of $K_{r}[w]$. Let the differential polynomial be defined by

$$F\bigl(w,w',\ldots,w^{(m)}\bigr):=\sum _{r\in J}a_{r}K_{r}[w], $$

where *J* is a finite index set, and $a_{r}$ are constants, then $\deg F(w,w',\ldots,w^{(m)}):=\max_{r\in J}\{d(r)\}$ is the degree of $F(w,w',\ldots,w^{(m)})$.

Consider the following differential equation:

16 $$ F\bigl(w,w',\ldots,w^{(m)}\bigr)=cw^{n}+d, $$

where $n\in{\mathbb {N}}^{*}$, $c\neq0$, *d* are constants.

Set $p, q\in{\mathbb {N}}^{*}$, and the meromorphic solutions *w* of equation (16) have at least one pole. If equation (16) has exactly *p* distinct meromorphic solutions, and their multiplicity of the pole at $z=0$ is *q*, then equation (16) is said to satisfy the $\langle p,q \rangle$ condition. It might not be easy to show that the $\langle p,q \rangle$ condition of equation (16) holds, so we need the weak $\langle p,q \rangle $ condition as follows.

Inserting the Laurent series

17 $$ w(z)=\sum_{\lambda=-q}^{\infty}\beta_{\lambda}z^{\lambda},\quad \beta _{-q}\neq0, q>0, $$

into equation (16), we can determine exactly *p* different Laurent singular parts:

$$\sum_{\lambda=-q}^{-1}\beta_{\lambda}z^{\lambda}, $$

then equation (16) is said to satisfy the weak $\langle p,q \rangle$ condition.

Given two complex numbers $\nu_{1}$, $\nu_{2}$ such that $\operatorname{Im} \frac{\nu_{1}}{\nu_{2}} >0$, and let *L* be the discrete subset $L[2\nu_{1}, 2\nu_{2}]:=\{\nu\mid \nu=2a_{1}\nu_{1}+2a_{2}\nu_{2}, a_{1},a_{2}\in\mathbb {Z}\}$, and *L* is isomorphic to ${\mathbb {Z}}\times{\mathbb {Z}}$. Let the discriminant $\varDelta =\varDelta (b_{1},b_{2}):=b_{1}^{3}-27b_{2}^{2}$ and

$$l_{n}=l_{n}(L):=\sum_{\nu\in L\setminus\{0\}} \frac{1}{\nu^{n}}. $$

A meromorphic function $\wp(z):=\wp(z,g_{2},g_{3})$ with double periods $2\nu_{1}$, $2\nu_{2}$, which satisfies the following equation:

$$\bigl(\wp'(z)\bigr)^{2}=4 \wp(z)^{3}-g_{2} \wp(z)-g_{3}, $$

in which $g_{2}=60l_{4}$, $g_{3}=140l_{6}$, and $\varDelta (g_{2},g_{3})\neq0$, is called the Weierstrass elliptic function and satisfies an addition formula [28] as follows:

$$\wp(z-z_{0})= \frac{1}{4} \biggl[\frac{\wp'(z)+\wp'(z_{0})}{\wp(z)-\wp(z_{0})} \biggr]^{2}-\wp (z)-\wp(z_{0}). $$

If a meromorphic function *g* is a rational function of *z*, or a rational function of $e^{\alpha z}$, $\alpha\in{\mathbb {C}}$, or an elliptic function, then we say that *g* belongs to the class *W*.

In 2009, Eremenko *et al.* [29] studied the *m*th-order Briot-Bouquet equation (BBEq)

$$F\bigl(w,w^{(m)}\bigr)=\sum_{j=0}^{n}F_{j}(w) \bigl(w^{(m)}\bigr)^{j}=0, $$

where $F_{j}(w)$ are constant coefficients polynomials, $m\in{\mathbb {N}}^{*}$. For the *m*th-order BBEq, we have the following lemma.

#### Lemma 2.1

([28, 30, 31])

*Let* $m, n, p, h\in{\mathbb {N}}^{*}$, $\deg F(w,w^{(m)}) < n $, *and a* *mth*-*order BBEq*

$$F\bigl(w, w^{(m)}\bigr)=cw^{n}+d $$

*satisfies the weak* $\langle p,q \rangle$ *condition*, *then the meromorphic solutions* $w\in W$. *Supposing for some values of the parameters the solution* *w* *exists*, *then any other meromorphic solutions will be one parameter family* $w(z+z_{0})$, $z_{0}\in{\mathbb {C}}$. *In addition*, *every elliptic solution* *w* *with a pole at* $z=0$ *is expressed as*

18 $$\begin{aligned} w(z) =& \sum_{i=1}^{h-1}\sum _{j=2}^{q}\frac{(-1)^{j}\beta _{-ij}}{(j-1)!}\frac{d^{j-2}}{dz^{j-2}} \biggl(\frac{1}{4}\biggl[\frac{\wp'(z)+D_{i}}{\wp(z)-B_{i}}\biggr]^{2} -\wp(z) \biggr) \\ &{}+ \sum_{i=1}^{h-1}\frac{\beta_{-i1}}{2} \frac{\wp'(z)+D_{i}}{\wp(z)-B_{i}} +\sum_{j=2}^{q} \frac{(-1)^{j}\beta_{-hj}}{(j-1)!}\frac {d^{j-2}}{dz^{j-2}}\wp (z)+\beta_{0}, \end{aligned}$$

*where* $\beta_{-ij}$ *are determined by* (17), $\sum_{i=1}^{h}\beta _{-i1}=0$ *and* $D_{i}^{2}=4B_{i}^{3}-g_{2}B_{i}-g_{3}$.

*Every rational function solution* $w:=R(z)$ *is expressed as*

19 $$ R(z)=\sum_{i=1}^{h}\sum _{j=1}^{q}\frac{\beta_{ij}}{(z-z_{i})^{j}}+ \beta_{0}, $$

*which has* *h* (≤*p*) *distinct poles of multiplicity* *q*.

*Every simply periodic solution* $w:=R(\vartheta)$ *is a rational function of* $\vartheta=e^{\alpha z}$ ($\alpha\in{\mathbb {C}}$), *and is expressed as*

20 $$ R(\vartheta)=\sum_{i=1}^{h}\sum _{j=1}^{q}\frac{\beta_{ij}}{(\vartheta -\vartheta_{i})^{j}}+ \beta_{0}, $$

*which has* *h* (≤*p*) *distinct poles of multiplicity* *q*.

By the above definitions and lemma, we now present the complex method.

Step 1. Insert the transformation $T: u(x,y,t)\rightarrow w(z)$ defined by $(x,y,t)\rightarrow z $ into a given PDE to yield a nonlinear ODE.

Step 2. Insert (17) into the ODE to determine whether the weak $\langle p,q \rangle$ condition holds.

Step 3. Insert the indeterminate solutions introduced in Lemma 2.1 into the ODE, and then get meromorphic solutions of the ODE with a pole at $z=0$.

Step 4. Obtain meromorphic solutions $w(z-z_{0})$ by Lemma 2.1 and the addition formula.

Step 5. Inserting the inverse transformation $T^{-1}$ into the meromorphic solutions, we get the exact solutions for the original PDE.

## Symmetries and symmetry reduction

### Symmetries

In order to find the symmetry $\sigma=\sigma(x,y,s,t,u)$ of equation (4), we set

21 $$ \sigma=au_{x}+bu_{y}+cu_{s}+du_{t}+eu+f, $$

where *u* is the solution of equation (4), *a*, *b*, *c*, *d*, *e*, *f* are unknown functions of real variables *x*, *y*, *s*, *t*. According to Lie group analysis [21, 22], *σ* satisfies

22 $$ \sigma_{t}+a_{1}\sigma^{2}u_{x}+a_{1}u^{2} \sigma_{x}+a_{2}\sigma_{xxx}+a_{3}\sigma _{xyy}+a_{4}\sigma_{xss}+a_{5}\sigma u_{x}+a_{5}u\sigma_{x}+a_{6}\sigma _{xxt}=0. $$

Substituting equation (21) into equation (22), we have a new differential equation, where

23 $$ a_{2}u_{xxx}=-a_{1}u^{2}u_{x}-a_{3}u_{xyy}-a_{4}u_{xss}-a_{5}u u_{x}-a_{6}u_{xxt}-u_{t}. $$

By equation (21), equation (22) and equation (23), we have

24 $$ \begin{aligned} &a=c_{5},\qquad b=(c_{2}s+c_{3}), \qquad c=\biggl(c_{4}-\frac{a_{4}}{a_{3}}c_{2}y\biggr), \\ &d=c_{1},\qquad e=0,\qquad f=0, \end{aligned} $$

where $c_{i}$ ($i=2,3,4,5$) are real constants. Substituting equations (24) into equation (21), we achieve the symmetry of the gZK equation,

25 $$ \sigma=c_{5}u_{x}+(c_{2}s+c_{3})u_{y}+ \biggl(c_{4}-\frac{a_{4}}{a_{3}}c_{2}y\biggr)u_{s}+c_{1}u_{t}. $$

In order to find the symmetry $\sigma=\sigma(x,y,s,t,u)$ of equation (3), we set

26 $$ \sigma=au_{x}+bu_{y}+cu_{s}+du_{t}+eu+f. $$

Here *u* is the solution of equation (3), *a*, *b*, *c*, *d*, *e*, *f* are unknown functions of real variables *x*, *y*, *s*, *t*. According to Lie group analysis, *σ* satisfies

27 $$ \sigma_{xxxs}-4\sigma_{xt}+4u_{x} \sigma_{xs}+4u_{xs}\sigma _{x}+2u_{xx} \sigma_{s}+2u_{s}\sigma_{xx}+3 \sigma_{yy}=0. $$

Substituting equation (26) into equation (27), we have a new differential equation, where

28 $$ u_{xxxs}=4u_{xt}-4u_{x}u_{xs}-2u_{xx}u_{s}-3u_{yy}. $$

By equation (26), equation (27) and equation (28), we have

29 $$ \begin{aligned} &a=c_{1}x+c_{2},\qquad b=c_{3}y+c_{4}, \qquad c=(2c_{3}-3c_{1})s+\rho(t), \\ &d=(2c_{3}-c_{1})t+c_{5},\qquad e=c_{1},\qquad f=\rho'(t)x+\frac{2}{3} \rho''(t)y^{2}+\tau (t)y+\psi(t), \end{aligned} $$

where $c_{i}$ ($i=1,2,\ldots,5$) are real constants, $\rho(t)$, $\tau(t)$, $\psi(t) $ are arbitrary real functions of *t*. Substituting equations (29) into equation (26), we achieved the symmetry of YTSF equation

30 $$\begin{aligned} \sigma =&(c_{1}x+c_{2})u_{x}+(c_{3}y+c_{4})u_{y}+ \bigl((2c_{3}-3c_{1})s+\rho(t)\bigr)u_{s} \\ &{}+\bigl((2c_{3}-c_{1})t+c_{5} \bigr)u_{t}+c_{1}u+\rho'(t)x+ \frac{2}{3}\rho''(t)y^{2}+\tau (t)y+ \psi(t). \end{aligned}$$

### Symmetry reduction

By solving the characteristic equation (25) of *σ*

31 $$ \frac{dx}{c_{5}}=\frac{dy}{c_{2}s+c_{3}}=\frac{ds}{c_{4}-\frac {a_{4}}{a_{3}}c_{2}y}=\frac{dt}{c_{1}}= \frac{du}{0}, $$

we find different symmetry reduced equations. Without loss of generality, we have two reduced equations as follows.

Setting $c_{1}=c_{3}=c_{4}=c_{5}=0$, $c_{2}=1$, we have the first similarity solution of equation (4)

32 $$ u=\varphi(\xi,\eta), $$

where $\xi=x+t$, $\eta=\frac{y^{2}}{2a_{3}}+\frac{s^{2}}{2a_{4}}$. Substituting equation (32) into equation (4), we have the first symmetry reduced equation of equation (4)

33 $$ \varphi_{\xi}+a_{1}\varphi^{2} \varphi_{\xi}+(a_{2}+a_{3})\varphi_{\xi\xi\xi }+2 \varphi_{\xi\eta\eta}+a_{5}\varphi\varphi_{\xi}=0. $$

Setting $c_{1}=c_{2}=0$, $c_{3}=c_{4}=c_{5}=1$, solving $\sigma=0$, we have the second similarity solution of equation (4)

34 $$ u=\varphi(\xi,\eta), $$

where $\xi=x+y$, $\eta=s$. Substituting equation (34) into equation (4), we have the second symmetry reduced equation of equation (4)

$$a_{1}\varphi^{2}\varphi_{\xi}+(a_{2}+a_{3}) \varphi_{\xi\xi\xi}+a_{4}\varphi_{\xi \eta\eta}+a_{5} \varphi\varphi_{\xi}=0. $$

By solving the characteristic equation (30) of *σ*

35 $$\begin{aligned} \begin{aligned}[b] \frac{dx}{c_{1}x+c_{2}}&=\frac{dy}{c_{3}y+c_{4}}=\frac{ds}{(2c_{3}-3c_{1})s+\rho(t)} \\ &=\frac{dt}{(2c_{3}-c_{1})t+c_{5}}=\frac{du}{c_{1}u+\rho'(t)x+\frac{2}{3}\rho ''(t)y^{2}+\tau(t) y+\psi(t)}, \end{aligned} \end{aligned}$$

we obtain symmetry reduction of equation (3). Without loss of generality, we have two reduced equations as follows.

Setting $c_{1}=c_{3}=c_{4}=0$, $c_{2}=c_{5}=1$, $\rho(t)=1$, solving $\sigma=0$, we have the first similarity solution of equation (3)

36 $$ u=\varphi(\xi,\eta,y)- \int\bigl(\tau(t)y+\psi(t)\bigr)\,dt, $$

where $\xi=x-t$, $\eta=s-t$. Substituting equation (36) into equation (3), we have the first symmetry reduced equation of equation (3)

37 $$ \varphi_{\xi\xi\xi\eta}+4\varphi_{\xi\xi}+4\varphi_{\xi\eta}+4\varphi _{\xi}\varphi_{\xi\eta}+2\varphi_{\xi\xi} \varphi_{\eta}+3\varphi_{yy}=0. $$

Setting $c_{1}=c_{2}=c_{3}=c_{5}=0$, $c_{4}=1$, $\rho(t)=\tau(t)=0$, solving $\sigma=0$, we have the second similarity solution of equation (3)

38 $$ u=\varphi(x,s,t)-\psi(t)y. $$

Substituting equation (38) into equation (3), we have the second symmetry reduced equation of equation (3)

$$\varphi_{xxxs}+4\varphi_{x}\varphi_{xs}+2 \varphi_{xx}\varphi_{s}-4\varphi _{xt}=0. $$

## Exact solutions

### Exact solutions of gZK equation via the $\exp(-\phi (z))$-expansion method

Substituting the traveling wave transform

$$\varphi(\xi,\eta)=w(z),\quad z=k\xi+l\eta, $$

into equation (33), then integrating it with respect to *z*, we obtain

39 $$ \bigl((a_{2}+a_{3}){k}^{2}+2l^{2} \bigr)w''+w + \frac{a_{5}}{2}w^{2}+ \frac {a_{1}}{3}w^{3}-\gamma=0, $$

where *γ* is the integration constant which can be determined later.

Taking the homogeneous balance between $w''$ and $w^{3}$ in equation (39) yields

40 $$ w(z)=C_{0}+C_{1}\exp\bigl(-\phi(z)\bigr), $$

where $C_{1}\neq0$, $C_{0}$ are constants to be determined, whereas *δ* and *μ* are arbitrary constants.

Substitute *w*, $w^{2}$, $w^{3}$, $w''$ into equation (39) and equate the coefficients of $\exp(-\phi(z))$ to zero, then

$$\begin{aligned}& \frac{1}{3} a_{1} {C_{0}}^{3}+ \frac{1}{2} a_{5} {C_{0}}^{2}+C_{0}+2 C_{1} {l}^{2 }\delta\mu+C_{1} {k}^{2}a_{2} \delta\mu+C_{1} {k}^{2}a_{3} \delta\mu-\gamma=0, \\& C_{1} a_{2} {k}^{2}{\delta}^{2}+C_{1} a_{3} {k}^{2}{\delta}^{2}+2 C_{1} {l}^{2}{\delta}^{2}+2 C_{1} a_{2} {k}^{2}\mu+2 C_{1} a_{ 3} {k}^{2} \mu \\& \quad {}+{C_{0}}^{2}C_{1} a_{1}+4 C_{1} {l}^{2}\mu+C_{0} C_{ 1} a_{5}+C_{1}=0, \\& \frac{1}{2} a_{5} {C_{1}}^{2}+a_{1} C_{0} {C_{1}}^{2}+6 C_{1} {l}^{2} \delta+3 C_{1} {k}^{2}a_{2} \delta+3 C_{1} {k}^{2}a_{3} \delta=0, \\& 4 C_{1} {l}^{2}+\frac{1}{3} a_{1} {C_{1}}^{3}+2 C_{1} {k}^{2}a_{2}+2 C_{1} {k}^{2}a_{3}=0. \end{aligned}$$

Solving the above algebraic equations, we obtain

41 $$ \begin{aligned} &\gamma=-\frac{\sqrt{-2 a_{1} ((\delta^{2}-4\mu) ( a_{2} {k}^{2}+a_{3} {k}^{2}+2 {l}^{2} )-2)}((\delta^{2}-4\mu) ( a_{2} {k}^{2}+a_{3} {k}^{2}+2 {l}^{2} )+1)}{6a_{1}}, \\ &C_{1}= \sqrt{\frac{-6 ( a_{2} {k}^{2}+a_{3} {k}^{2}+2 {l}^{2} )}{a_{1}}}, \\ &C_{0}=\frac{\sqrt{-6 a_{1} ( a_{2} {k}^{2}+a_{3} {k}^{2}+2 {l}^{2} )}\delta-\sqrt{2 a_{1} (2-(\delta^{2}-4\mu) ( a_{2} {k}^{2}+a_{3} {k}^{2}+2 {l}^{2} ))}}{2a_{1}}, \end{aligned} $$

where *μ* and *δ* are arbitrary constants.

Substituting equations (41) into equation (40) yields

42 $$\begin{aligned} w(z) =&\frac{\sqrt{-6 a_{1} ( a_{2} {k}^{2}+a_{3} {k}^{2}+2 {l}^{2} )}\delta-\sqrt{2 a_{1} (2-(\delta^{2}-4\mu) ( a_{2} {k}^{2}+a_{3} {k}^{2}+2 {l}^{2} ))}}{2a_{1}} \\ &{}+\sqrt{\frac{-6 ( a_{2} {k}^{2}+a_{3} {k}^{2}+2 {l}^{2} )}{a_{1}}} \exp\bigl(-\phi(z)\bigr). \end{aligned}$$

We apply equation (9) to equation (15) into equation (42), respectively, to get traveling wave solutions of the gZK equation as follows.

When $\delta^{2}-4\mu>0$, $\mu\neq0$,

$$\begin{aligned}& \begin{aligned} w_{11}(z)=&{}\frac{\sqrt{-6 a_{1} ( a_{2} {k}^{2}+a_{3} {k}^{2}+2 {l}^{2} )}\delta-\sqrt{2 a_{1} (2-(\delta^{2}-4\mu) ( a_{2} {k}^{2}+a_{3} {k}^{2}+2 {l}^{2} ))}}{2a_{1}} \\ &{}-\sqrt{\frac{-6 ( a_{2} {k}^{2}+a_{3} {k}^{2}+2 {l}^{2} )}{a_{1}}} \frac{2\mu}{\sqrt{(\delta^{2}-4\mu)}\tanh(\frac{\sqrt{\delta ^{2}-4\mu}}{2}(z+c)+\delta)}, \end{aligned} \\& \begin{aligned} w_{12}(z)={}&\frac{\sqrt{-6 a_{1} ( a_{2} {k}^{2}+a_{3} {k}^{2}+2 {l}^{2} )}\delta-\sqrt{2 a_{1} (2-(\delta^{2}-4\mu) ( a_{2} {k}^{2}+a_{3} {k}^{2}+2 {l}^{2} ))}}{2a_{1}} \\ &{}-\sqrt{\frac{-6 ( a_{2} {k}^{2}+a_{3} {k}^{2}+2 {l}^{2} )}{a_{1}}} \frac{2\mu}{\sqrt{(\delta^{2}-4\mu)}\coth(\frac{\sqrt{\delta ^{2}-4\mu}}{2}(z+c)+\delta)}. \end{aligned} \end{aligned}$$

When $\delta^{2}-4\mu<0$, $\mu\neq0$,

$$\begin{aligned}& \begin{aligned} w_{13}(z)={}&\frac{\sqrt{-6 a_{1} ( a_{2} {k}^{2}+a_{3} {k}^{2}+2 {l}^{2} )}\delta-\sqrt{2 a_{1} (2-(\delta^{2}-4\mu) ( a_{2} {k}^{2}+a_{3} {k}^{2}+2 {l}^{2} ))}}{2a_{1}} \\ &{}+\sqrt{\frac{-6 ( a_{2} {k}^{2}+a_{3} {k}^{2}+2 {l}^{2} )}{a_{1}}} \frac{2\mu}{\sqrt{(4\mu-\delta^{2})}\tan(\frac{\sqrt{4\mu -\delta^{2}}}{2}(z+c)-\delta)}, \end{aligned} \\& \begin{aligned} w_{14}(z)={}&\frac{\sqrt{-6 a_{1} ( a_{2} {k}^{2}+a_{3} {k}^{2}+2 {l}^{2} )}\delta-\sqrt{2 a_{1} (2-(\delta^{2}-4\mu) ( a_{2} {k}^{2}+a_{3} {k}^{2}+2 {l}^{2} ))}}{2a_{1}} \\ &{}+\sqrt{\frac{-6 ( a_{2} {k}^{2}+a_{3} {k}^{2}+2 {l}^{2} )}{a_{1}}} \frac{2\mu}{\sqrt{(4\mu-\delta^{2})}\cot(\frac{\sqrt{4\mu -\delta^{2}}}{2}(z+c)-\delta)}. \end{aligned} \end{aligned}$$

When $\delta^{2}-4\mu>0$, $\mu=0$, $\delta\neq0$,

$$\begin{aligned} w_{15}(z) =&\frac{\sqrt{-6 a_{1} ( a_{2} {k}^{2}+a_{3} {k}^{2}+2 {l}^{2} )}\delta-\sqrt{2 a_{1} (2-\delta^{2} ( a_{2} {k}^{2}+a_{3} {k}^{2}+2 {l}^{2} ))}}{2a_{1}} \\ &{}+\sqrt{\frac{-6 ( a_{2} {k}^{2}+a_{3} {k}^{2}+2 {l}^{2} )}{a_{1}}} \frac{\delta}{\exp(\delta(z+c))-1}. \end{aligned}$$

When $\delta^{2}-4\mu=0$, $\mu\neq0$, $\delta\neq0$,

$$w_{16}(z)=\sqrt{\frac{-3 ( a_{2} {k}^{2}+a_{3} {k}^{2}+2 {l}^{2} )}{2a_{1}}}\delta-\frac{1}{\sqrt{a_{1}}}-\sqrt{ \frac{-6 ( a_{2} {k}^{2}+a_{3} {k}^{2}+2 {l}^{2} )}{a_{1}}} \frac{\delta ^{2}(z+c)}{2(\delta(z+c)+2)}. $$

When $\delta^{2}-4\mu=0$, $\mu=0$, $\delta=0$,

$$w_{17}(z)=-\frac{1}{\sqrt{a_{1}}}+\sqrt{\frac{-6 ( a_{2} {k}^{2}+a_{3} {k}^{2}+2 {l}^{2} )}{a_{1}}} \frac{1}{z+c}. $$

### Exact solutions of gZK equation via the complex method

Inserting (17) into equation (39) we have $p=2$, $q=1$, $\beta_{{-1}}=\pm\sqrt{\frac{-6 ( a_{2} {k}^{2}+a_{3} {k}^{2}+2 {l}^{2} )}{a_{1}}}$, $\beta_{{0}}=-\frac{a_{5}}{2a_{1}}$, $\beta_{{1}}=-\frac{a_{5}^{2}}{24a_{1}^{2}}\sqrt {\frac{-6a_{1}}{ a_{2} {k}^{2}+a_{3} {k}^{2}+2 {l}^{2}}}$, $\beta _{{2}}=-\frac{12a_{1}^{2}\gamma-a_{5}^{3}+6a_{1}a_{5}}{48a_{1}^{2}( a_{2} {k}^{2}+a_{3} {k}^{2}+2 {l}^{2})}$ and $\beta_{3}$ is an arbitrary constant.

Therefore, equation (39) is a second order BBEq and satisfies the weak $\langle2,1 \rangle$ condition. Hence, by Lemma 2.1, we see that meromorphic solutions of equation (39) belong to *W*. We will show meromorphic solutions of equation (39) in the following.

By (19), we infer that the indeterminate rational solutions of equation (39) are

$$R_{1}(z)=\frac{\beta_{11}}{z} +\frac{\beta_{12}}{z-z_{1}}+\beta_{10}, $$

with a pole at $z=0$.

Substituting $R_{1}(z)$ into equation (39), we have

$$R_{1,1}(z)=\pm\sqrt{\frac{-6 ( a_{2} {k}^{2}+a_{3} {k}^{2}+2 {l}^{2} )}{a_{1}}}\frac{1}{z}- \frac{a_{5}}{2a_{1}}, $$

where $a_{5}^{2}=4a_{1}$ and $9a_{1}\gamma^{2}=1$;

$$R_{1,2}(z)=\pm\sqrt{\frac{-6 ( a_{2} {k}^{2}+a_{3} {k}^{2}+2 {l}^{2} )}{a_{1}}}\biggl(\frac{1}{z}- \frac{1}{z-z_{1}}-\frac {1}{z_{1}}\biggr)-\frac{a_{5}}{2a_{1}}, $$

where $k=\sqrt{\frac{4a_{1}z_{1}^{2}-a_{5}^{2}z_{1}^{2}-48l^{2}a_{1}}{24a_{1}(a_{2}+a_{3})}}$ and $\gamma=(a_{5}^{3}-6a_{1}a_{5}+(a_{5}^{2}-4a_{1})^{\frac{3}{3}})z_{1}^{3}$.

So the rational solutions of equation (39) are

$$w_{r,1}(z)=\pm\sqrt{\frac{-6 ( a_{2} {k}^{2}+a_{3} {k}^{2}+2 {l}^{2} )}{a_{1}}}\frac{1}{z-z_{0}}- \frac{a_{5}}{2a_{1}} $$

and

$$w_{r,2}(z)=\pm\sqrt{\frac{-6 ( a_{2} {k}^{2}+a_{3} {k}^{2}+2 {l}^{2} )}{a_{1}}}\biggl(\frac{1}{z-z_{0}}- \frac{1}{z-z_{0}-z_{1}}-\frac {1}{z_{1}}\biggr)-\frac{a_{5}}{2a_{1}}, $$

where $z_{0}\in{\mathbb {C}}$, $z_{1}\neq0$. $a_{5}^{2}=4a_{1}$, $9a_{1}\gamma^{2}=1$ in the former case, or $k=\sqrt{\frac {4a_{1}z_{1}^{2}-a_{5}^{2}z_{1}^{2}-48l^{2}a_{1}}{24a_{1}(a_{2}+a_{3})}}$, $\gamma =(a_{5}^{3}-6a_{1}a_{5}+(a_{5}^{2}-4a_{1})^{\frac{3}{3}})z_{1}^{3}$ in the latter case.

To obtain simply periodic solutions, let $\vartheta=e^{\alpha z}$, and substitute $w=R(\vartheta)$ into equation (39), then

43 $$ \bigl((a_{2}+a_{3}){k}^{2}+2l^{2} \bigr)\alpha^{2}\bigl(\vartheta R'+\vartheta^{2}R'' \bigr)+R + \frac{a_{5}}{2}R^{2}+\frac{a_{1}}{3}R^{3}- \gamma=0. $$

Substituting

$$R_{2}(z)=\frac{\beta_{21}}{\vartheta-1} +\frac{\beta_{22}}{(\vartheta -\vartheta_{1})}+\beta_{20} $$

into equation (43), we obtain

44 $$ R_{2,1}(z)=\pm\sqrt{\frac{-6 ( a_{2} {k}^{2}+a_{3} {k}^{2}+2 {l}^{2} )}{a_{1}}}\alpha\biggl(\frac{1}{\vartheta-1} +\frac{1}{2}\biggr)-\frac {a_{5}}{2a_{1}} $$

and

45 $$ R_{2,2}(z)=\pm\sqrt{\frac{-6 ( a_{2} {k}^{2}+a_{3} {k}^{2}+2 {l}^{2} )}{a_{1}}}\alpha\biggl(\frac{1}{\vartheta-1} -\frac{\vartheta _{1}}{\vartheta-\vartheta_{1}}-\frac{\vartheta_{1}+1}{2(\vartheta_{1}-1)}\biggr)-\frac {a_{5}}{2a_{1}}, $$

where $\gamma = \frac{a_{5}(a_{5}^{2}-6a_{1})}{12a_{1}^{2}}$, $l = \frac{1}{2\alpha} \sqrt{ \frac{4a_{1}-a_{5}^{2}-2a_{1}k^{2}\alpha ^{2}(a_{2}+a_{3})}{a_{1}}}$ in the former case, or $\gamma = \frac{\sqrt {3}z_{1}(z_{1}+1)(4a_{1}-a_{5}^{2})^{\frac{3}{2}}}{(z_{1}^{2}+10z_{1}+1)^{\frac {3}{2}}a_{1}^{2}} +\frac{a_{5}(a_{5}^{2}-6a_{1})}{12a_{1}^{2}}$, $k=-\sqrt{\frac{(4a_{1}-a_{5}^{2}-4a_{1}l^{2}\alpha ^{2})(z_{1}^{2}+1)+2(a_{5}^{2}-4a_{1}-20a_{1}l^{2}\alpha ^{2})z_{1}}{2a_{1}(z_{1}^{2}+10z_{1}+1)(a_{2}+a_{3})\alpha^{2}}}$ in the latter case.

Inserting $\vartheta=e^{\alpha z}$ into equation (44) and equation (45), we can get simply periodic solutions to equation (39) with a pole at $z=0$,

$$\begin{aligned}& w_{s0,1}(z) =\pm\sqrt{\frac{-3 ( a_{2} {k}^{2}+a_{3} {k}^{2}+2 {l}^{2} )}{2a_{1}}}\alpha\coth{ \frac{\alpha}{2}}z-\frac{a_{5}}{2a_{1}}, \\& w_{s0,2}(z) =\pm\sqrt{\frac{-3 ( a_{2} {k}^{2}+a_{3} {k}^{2}+2 {l}^{2} )}{2a_{1}}}\alpha\biggl(\coth{ \frac{\alpha}{2}}z-\coth{\frac {\alpha}{2}}(z-z_{1})-\coth{ \frac{\alpha}{2}}z_{1}\biggr) -\frac{a_{5}}{2a_{1}}, \end{aligned}$$

where $\gamma = \frac{a_{5}(a_{5}^{2}-6a_{1})}{12a_{1}^{2}}$, $l = \frac{1}{2\alpha} \sqrt{ \frac{4a_{1}-a_{5}^{2}-2a_{1}k^{2}\alpha ^{2}(a_{2}+a_{3})}{a_{1}}}$ in the former case, or $\gamma = \frac{\sqrt {3}z_{1} (z_{1}+1)(4a_{1}-a_{5}^{2})^{\frac{3}{2}}}{(z_{1}^{2}+10z_{1}+1)^{\frac {3}{2}}a_{1}^{2}}+\frac{a_{5}(a_{5}^{2}-6a_{1})}{12a_{1}^{2}}$, $k=-\sqrt{\frac{(4a_{1}-a_{5}^{2}-4a_{1}l^{2}\alpha ^{2})(z_{1}^{2}+1)+2(a_{5}^{2}-4a_{1}-20a_{1}l^{2}\alpha ^{2})z_{1}}{2a_{1}(z_{1}^{2}+10z_{1}+1)(a_{2}+a_{3})\alpha^{2}}}$ in the latter case.

So simply periodic solutions of equation (39) are

$$w_{s,1}(z) =\pm\sqrt{\frac{-3 ( a_{2} {k}^{2}+a_{3} {k}^{2}+2 {l}^{2} )}{2a_{1}}}\alpha\coth{ \frac{\alpha}{2}}(z-z_{0})-\frac{a_{5}}{2a_{1}} $$

and

$$\begin{aligned} w_{s,2}(z) =&\pm\sqrt{\frac{-3 ( a_{2} {k}^{2}+a_{3} {k}^{2}+2 {l}^{2} )}{2a_{1}}} \\ &{}\cdot\alpha\biggl(\coth{ \frac{\alpha}{2}}(z-z_{0})-\coth {\frac{\alpha}{2}}(z-z_{0}-z_{1})- \coth{\frac{\alpha}{2}}z_{1}\biggr)-\frac{a_{5}}{2a_{1}}, \end{aligned}$$

where $z_{0}\in{\mathbb {C}}$, $z_{1}\neq0$. $l=\frac{1}{2\alpha}\sqrt{\frac {4a_{1}-a_{5}^{2}-2a_{1}k^{2}\alpha^{2}(a_{2}+a_{3})}{a_{1}}}$, $\gamma=\frac {a_{5}(a_{5}^{2}-6a_{1})}{12a_{1}^{2}}$ in the former case, or $k=-\sqrt{\frac{(4a_{1}-a_{5}^{2}-4a_{1}l^{2}\alpha ^{2})(z_{1}^{2}+1)+2(a_{5}^{2}-4a_{1}-20a_{1}l^{2}\alpha ^{2})z_{1}}{2a_{1}(z_{1}^{2}+10z_{1}+1)(a_{2}+a_{3})\alpha^{2}}}$, $\gamma=\frac{\sqrt {3}z_{1}(z_{1}+1)(4a_{1}-a_{5}^{2})^{\frac{3}{2}}}{(z_{1}^{2}+10z_{1}+1)^{\frac {3}{2}}a_{1}^{2}}+\frac{a_{5}(a_{5}^{2}-6a_{1})}{12a_{1}^{2}}$ in the latter case.

From (18), we have the indeterminate relations for the elliptic solutions of equation (39) with a pole at $z=0$,

$$w_{d1}(z)=\frac{\beta_{-1}}{2}\frac{\wp'(z)+D_{1}}{\wp(z)-B_{1}} +\beta_{0}, $$

where $D_{1}^{2}=4B_{1}^{3}-g_{2}B_{1}-g_{3}$. Considering the results obtained above, we infer that $\beta_{0}=-\frac{a_{5}}{2a_{1}}$, $g_{3}=0$, $D_{1}=B_{1}=0$. So we obtain

$$w_{d1}(z)=\pm\sqrt{\frac{-3 ( a_{2} {k}^{2}+a_{3} {k}^{2}+2 {l}^{2} )}{2a_{1}}}\frac{\wp'(z)}{\wp(z)}- \frac{a_{5}}{2a_{1}}, $$

where $g_{3}=0$.

Thus, the elliptic function solutions of equation (39) are

$$w_{d}(z)=\pm\sqrt{\frac{-3 ( a_{2} {k}^{2}+a_{3} {k}^{2}+2 {l}^{2} )}{2a_{1}}}\frac{\wp'(z-z_{0},g_{2},0)}{\wp(z-z_{0},g_{2},0)}- \frac{a_{5}}{2a_{1}}, $$

where $z_{0}\in{\mathbb {C}}$, $g_{3}=0$, $g_{2}$ is arbitrary. Applying the addition formula, we can rewrite it as

$$\begin{aligned} w_{d}(z) =&\pm\sqrt{\frac{-3 ( a_{2} {k}^{2}+a_{3} {k}^{2}+2 {l}^{2} )}{2a_{1}}} \\ &{}\cdot{\frac{(-\wp+E)(4E\wp^{2}+(4E^{2}-g_{{2}})\wp+2F\wp' -Eg_{{2}})}{((12{E}^{2}-g_{{2}})\wp+4{E}^{3}-3Eg_{{2}})\wp'+(4\wp ^{3}+12E\wp^{2}-3 g_{{2}}\wp-Eg_{{2}})F}}-\frac{a_{5}}{2a_{1}}, \end{aligned}$$

where $g_{3}=0$, $F^{2}=4E^{3}-g_{2}E$, *E* and $g_{2}$ are arbitrary.

### Exact solutions of YTSF equation via the $\exp(-\phi (z))$-expansion method

Substituting the traveling wave transform

$$\varphi(\xi,\eta,y)=v(z),\quad z=k\xi+l\eta+ry, $$

into equation (37), then integrating it with respect to *z*, we obtain

46 $$ k^{3}lv''' + \bigl(4k^{2}+4kl+3r^{2}\bigr)v'+3k^{2}l \bigl(v'\bigr)^{2}+\gamma=0, $$

where *γ* is the integration constant which can be determine later.

Setting $w=v'$, equation (46) becomes

47 $$ k^{3}lw'' + \bigl(4k^{2}+4kl+3r^{2} \bigr)w+3k^{2}lw^{2}+\gamma=0. $$

Taking the homogeneous balance between $w''$ and $w^{2}$ in equation (47) yields

48 $$ w(z)=C_{0}+C_{1}\exp\bigl(-\phi(z)\bigr)+C_{2} \bigl(\exp\bigl(\phi(z)\bigr)\bigr)^{2}, $$

where $C_{2}\neq0$, $C_{i}$ ($i=0,1,2$) are constants to be determined, whereas *δ* and *μ* are arbitrary constants.

Substitute *w*, $w^{2}$, $w''$ into equation (47) and equate the coefficients of $\exp(-\phi(z))$ to zero, then

$$\begin{aligned}& {k}^{3}lC_{1} \delta\mu+2 {k}^{3}lC_{2} {\mu}^{2}+3 {k}^{2}l{C_{0}}^{2}+4 C_{0} {k}^{2}+4 C_{0} kl+3 C_{0} {r}^{2}+\gamma=0, \\& C_{1} l{k}^{3}{\delta}^{2}+6 C_{2} l{k}^{3}\delta\mu+2 C_{1} l{k}^{3}\mu+6 C_{0} C_{1} l{k}^{2}+4 C_{1} {k}^{2}+4 C_{1} lk+3 C_{1} {r}^{2}=0, \\& 4 C_{2} l{k}^{3}{\delta}^{2}+3 C_{1} l{k}^{3}\delta+8 C_{2} l{k}^{3}\mu +6 C_{0} C_{2} l{k}^{2}+3 {C_{1}}^{2}l{k}^{2}+4 C_{2} {k}^{2}+4 C_{2} lk+3 C_{2} {r}^{2}=0, \\& 10 C_{2} l{k}^{3}\delta+6 C_{1} C_{2} l{k}^{2}+2 C_{1} l{k}^{3}=0, \\& 3 {C_{2}}^{2}l{k}^{2}+6 C_{2} l{k}^{3}=0. \end{aligned}$$

Solving the above algebraic equations, we obtain

49 $$ \begin{aligned} &\gamma=-\frac{(\delta^{2}-4\mu)^{2}l^{2}k^{6}-(4lk+4k^{2}+3r^{2})^{2}}{12k^{2}l},\qquad C_{2}=-2k, \\ &C_{1}=-2k\delta,\qquad C_{0}=-\frac{lk^{3}\delta^{2}+8lk^{3}\mu+4lk+4k^{2}+3r^{2}}{6k^{2}l}, \end{aligned} $$

where *μ* and *δ* are arbitrary constants.

Substituting equations (49) into equation (48), yields

50 $$ w(z)=-\frac{lk^{3}\delta^{2}+8lk^{3}\mu+4lk+4k^{2}+3r^{2}}{6k^{2}l}-2k\delta\exp \bigl(-\phi(z)\bigr)-2k\bigl(\exp\bigl( \phi(z)\bigr)\bigr)^{2}. $$

We apply equation (9) to equation (15) into equation (50), respectively, to get traveling wave solutions of the YTSF equation as follows.

When $\delta^{2}-4\mu>0$, $\mu\neq0$,

$$\begin{aligned}& \begin{aligned} w_{21}(z)={}&{-}\frac{lk^{3}\delta^{2}+8lk^{3}\mu+4lk+4k^{2}+3r^{2}}{6k^{2}l}+ \frac {4k\delta\mu}{\sqrt{(\delta^{2}-4\mu)}\tanh(\frac{\sqrt{\delta^{2}-4\mu }}{2}(z+c)+\delta)} \\ &{}-\frac{8k\mu^{2}}{(\sqrt{(\delta^{2}-4\mu)}\tanh(\frac{\sqrt{\delta^{2}-4\mu }}{2}(z+c)+\delta))^{2}}, \end{aligned} \\& \begin{aligned} w_{22}(z)={}&{-}\frac{lk^{3}\delta^{2}+8lk^{3}\mu+4lk+4k^{2}+3r^{2}}{6k^{2}l}+ \frac {4k\delta\mu}{\sqrt{(\delta^{2}-4\mu)}\coth(\frac{\sqrt{\delta^{2}-4\mu }}{2}(z+c)+\delta)} \\ &{}-\frac{8k\mu^{2}}{(\sqrt{(\delta^{2}-4\mu)}\coth(\frac{\sqrt{\delta^{2}-4\mu }}{2}(z+c)+\delta))^{2}}. \end{aligned} \end{aligned}$$

When $\delta^{2}-4\mu<0$, $\mu\neq0$,

$$\begin{aligned}& \begin{aligned} w_{23}(z)={}&{-}\frac{lk^{3}\delta^{2}+8lk^{3}\mu+4lk+4k^{2}+3r^{2}}{6k^{2}l}- \frac {4k\delta\mu}{\sqrt{(\delta^{2}-4\mu)}\tan(\frac{\sqrt{\delta^{2}-4\mu }}{2}(z+c)-\delta)} \\ &{}-\frac{8k\mu^{2}}{(\sqrt{(\delta^{2}-4\mu)}\tan(\frac{\sqrt{\delta^{2}-4\mu }}{2}(z+c)-\delta))^{2}}, \end{aligned} \\& \begin{aligned} w_{24}(z)={}&{-}\frac{lk^{3}\delta^{2}+8lk^{3}\mu+4lk+4k^{2}+3r^{2}}{6k^{2}l}- \frac {4k\delta\mu}{\sqrt{(\delta^{2}-4\mu)}\cot(\frac{\sqrt{\delta^{2}-4\mu }}{2}(z+c)-\delta)} \\ &{}-\frac{8k\mu^{2}}{(\sqrt{(\delta^{2}-4\mu)}\cot(\frac{\sqrt{\delta^{2}-4\mu }}{2}(z+c)-\delta))^{2}}. \end{aligned} \end{aligned}$$

When $\delta^{2}-4\mu>0$, $\mu=0$, $\delta\neq0$,

$$w_{25}(z)=-\frac{lk^{3}\delta^{2}+4lk+4k^{2}+3r^{2}}{6k^{2}l}- \frac{2k\delta ^{2}}{\exp(\delta(z+c))-1}- \frac{2k\delta^{2}}{(\exp(\delta(z+c))-1)^{2}}. $$

When $\delta^{2}-4\mu=0$, $\mu\neq0$, $\delta\neq0$,

$$w_{26}(z)=-\frac{12lk^{3}\mu+4lk+4k^{2}+3r^{2}}{6k^{2}l}+ \frac{k\delta ^{3}(z+c)}{(\delta(z+c)+2)}- \frac{k\delta^{4}(z+c)^{2}}{2((\delta(z+c)+2))^{2}}. $$

When $\delta^{2}-4\mu=0$, $\mu=0$, $\delta=0$,

$$w_{27}(z)=-\frac{4lk+4k^{2}+3r^{2}}{6k^{2}l}-\frac{2k}{(z+c)^{2}}. $$

### Exact solutions of YTSF equation via the complex method

Inserting (17) into equation (47) we have $p=1$, $q=2$, $\beta_{{-2}}=-2k$, $\beta_{{-1}}=0$, $\beta_{{0}}=-\frac {4lk+4k^{2}+3r^{2}}{6k^{2}l}$, $\beta_{{1}}=0$, $\beta_{{2}}=-\frac {16k^{4}+32lk^{3}+(16l^{2}-12l\gamma+24r^{2})k^{2}+24lkr^{2}+9r^{4}}{120k^{5}l^{2}}$, and $\beta_{3}$ is an arbitrary constant.

Therefore, equation (47) is a second order BBEq and satisfies the weak $\langle1,2 \rangle$ condition. Hence, by Lemma 2.1, we see that meromorphic solutions of equation (47) belong to *W*. We will show meromorphic solutions of equation (47) in the following.

By (19), we deduce the indeterminacy rational solutions of equation (47) are

$$R_{1}(z)=\frac{\beta_{32}}{z^{2}} +\frac{\beta_{31}}{z}+ \beta_{30}, $$

with a pole at $z=0$.

Substituting $R_{1}(z)$ into equation (47), we get the following form:

$$R_{1}(z)=-\frac{2k}{z^{2}}-\frac{4lk+4k^{2}+3r^{2}}{6k^{2}l}, $$

where $\gamma=\frac{16k^{4}+32lk^{3}+(16l^{2}+24r^{2})k^{2}+24lkr^{2}+9r^{4}}{12k^{2}l}$.

So the rational solutions of equation (47) are

$$w_{r}(z)=-\frac{2k}{(z-z_{0})^{2}}-\frac{4lk+4k^{2}+3r^{2}}{6k^{2}l}, $$

where $\gamma=\frac {16k^{4}+32lk^{3}+(16l^{2}+24r^{2})k^{2}+24lkr^{2}+9r^{4}}{12k^{2}l}$, $z_{0}\in{\mathbb {C}}$.

To obtain simply periodic solutions, let $\vartheta=e^{\alpha z}$, and substitute $w=R(\vartheta)$ into equation (47), then we get

51 $$ k^{3}l\alpha^{2}\bigl(\vartheta R'+ \vartheta^{2}R''\bigr) + \bigl(4k^{2}+4kl+3r^{2}\bigr)R+3k^{2}lR^{2}+ \gamma=0. $$

Substituting

$$R_{2}(z)=\frac{\beta_{42}}{(\vartheta-1)^{2}} +\frac{\beta _{41}}{(\vartheta-1)}+ \beta_{40}, $$

into equation (51), we obtain

52 $$ R_{2}(z)=-\frac{2k\alpha^{2}}{(\vartheta-1)^{2}} -\frac{2k\alpha ^{2}}{(\vartheta-1)}- \frac{k\alpha^{2}}{6}-\frac{4lk+4k^{2}+3r^{2}}{6k^{2}l}, $$

where $\gamma=\frac{(4lk+4k^{2}+3r^{2})^{2}-(l\alpha^{2}k^{3})^{2}}{12k^{2}l}$. Substituting $\vartheta=e^{\alpha z}$ into equation (52), we can obtain simply periodic solutions of equation (47),

$$\begin{aligned} w_{s0}(z) =&-\frac{2k\alpha^{2}}{(e^{\alpha z}-1)^{2}} -\frac{2k\alpha ^{2}}{(e^{\alpha z}-1)}- \frac{k\alpha^{2}}{6}-\frac{4lk+4k^{2}+3r^{2}}{6k^{2}l} \\ =& -\frac{2k\alpha^{2}e^{\alpha z}}{(e^{\alpha z}-1)^{2}}-\frac{k\alpha ^{2}}{6}-\frac{4lk+4k^{2}+3r^{2}}{6k^{2}l} \\ =&-\frac{k\alpha^{2}}{2} \coth^{2}\frac{\alpha z}{2}+ \frac{k\alpha ^{2}}{3}-\frac{4lk+4k^{2}+3r^{2}}{6k^{2}l}, \end{aligned}$$

with a pole at $z=0$.

Therefore the simply periodic solutions of equation (47) are

$$w_{s}(z)=-\frac{k\alpha^{2}}{2} \coth^{2}\frac{\alpha(z-z_{0})}{2} +\frac {k\alpha^{2}}{3}-\frac{4lk+4k^{2}+3r^{2}}{6k^{2}l}, $$

where $\gamma=\frac{(4lk+4k^{2}+3r^{2})^{2}-(l\alpha^{2}k^{3})^{2}}{12k^{2}l}$, $z_{0}\in{\mathbb {C}}$.

From (18), we can express the elliptic solutions of equation (47) as

$$w_{d0}(z)=\beta_{-2}\wp(z)+\beta_{0}, $$

with a pole at $z=0$.

Substituting $w_{d0}(z)$ into equation (47), we obtain

$$w_{d0}(z)=-2k\wp(z)-\frac{4lk+4k^{2}+3r^{2}}{6k^{2}l}, $$

where $g_{2}=\frac{16k^{4}+32lk^{3}+(16l^{2}-12l\gamma +24r^{2})k^{2}+24lkr^{2}+9r^{4}}{12k^{6}l^{2}}$, $g_{3}$ is arbitrary.

Therefore, the elliptic solutions of equation (47) are

$$w_{d}(z)=-2k\wp(z-z_{0})-\frac{4lk+4k^{2}+3r^{2}}{6k^{2}l}, $$

in which $z_{0}\in{\mathbb {C}}$. Applying the addition formula, we can rewrite it as

$$w_{d}(z)=-2k\biggl(-\wp(z)+\frac{1}{4}\biggl( \frac{\wp'(z)+C}{\wp (z)-D}\biggr)^{2}\biggr)+2kD-\frac{4lk+4k^{2}+3r^{2}}{6k^{2}l}, $$

where $g_{2}=\frac{16k^{4}+32lk^{3}+(16l^{2}-12l\gamma +24r^{2})k^{2}+24lkr^{2}+9r^{4}}{12k^{6}l^{2}}$, $C^{2}=4D^{3}-g_{2}D-g_{3}$, $g_{3}$ is arbitrary.

### Comparison

Implementing the $\exp(-\phi(z))$-expansion method, we found seven solutions for the gZK and YSFT equation, respectively. Using the complex method, we found five solutions for the gZK equation and three solutions for the YSFT equation. Rational solutions $w_{17}(z)$ and $w_{27}(z)$ are obtained via the $\exp(-\phi(z))$-expansion method, and $W_{r,1}(z)$ and $W_{r}(z)$ are obtained via the complex method. If we let $c=-z_{0}$, then $w_{17}(z)$ is equivalent to $W_{r,1}(z)$, and $w_{27}(z)$ is equivalent to $W_{r}(z)$. For getting rational solutions, these two methods are in good agreement. Rational solutions $W_{r,2}(z)$ and simply periodic solutions $W_{s,2}(z)$ and $W_{s}(z)$ are new and cannot be degenerated successively through elliptic function solutions. From the results, we can find more solutions by the $\exp(-\phi(z))$-expansion method, whereas we can obtain elliptic function solutions just by the complex method. These two methods are very useful tools in finding the exact solutions of NLEEs.

## Computer simulations

In this section, we illustrate some results by the computer simulations. We carry out further analysis to the properties of simply periodic solutions $W_{s,2}(z)$ and $W_{s}(z)$ as in Figures 1 and 2.

1. By employing the complex method, we are capable to obtain simply periodic solutions $W_{s,1}(z)$ and $W_{s,2}(z)$ of the gZK equation. The solutions $W_{s,1}(z)$ and $W_{s,2}(z)$ come from hyperbolic function. Figure 1 shows the shape of solutions $W_{s,2}(z)$ for $k=1$, $l=1$, $\alpha=1$, $a_{1}=-6$, $a_{2}=1$, $a_{3}=1$, $a_{5}=-24$, and $z_{1}=1$ within the interval $-2\pi\leq\xi, \eta\leq2\pi$. Note that they have two distinct generation poles which are showed by Figure 1.

**Figure 1:**
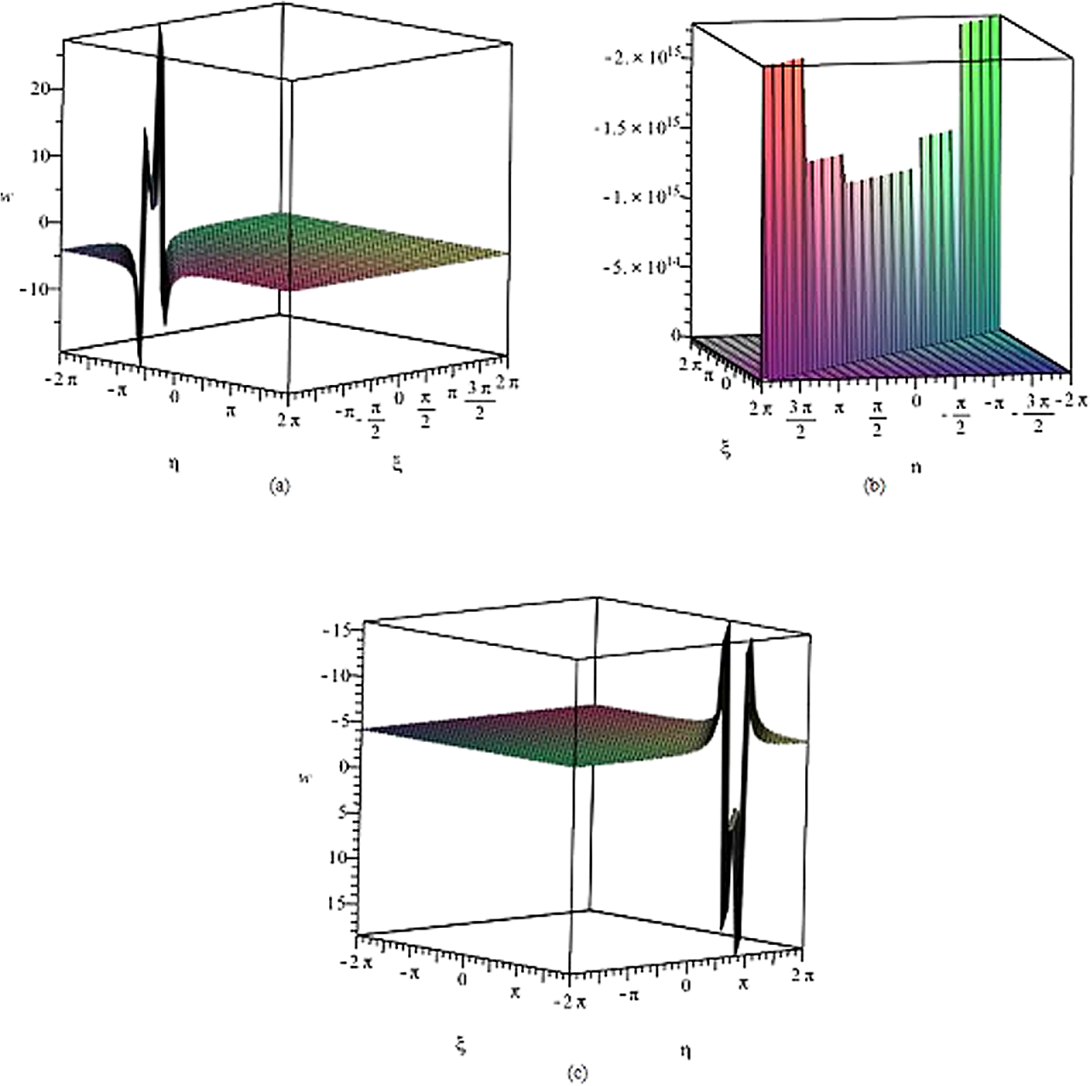
**The solution of the gZK equation corresponding to** $\pmb{W_{s,2}(z)}$ **.** **(a)** $z_{0}=-8$, **(b)** $z_{0}=0$, **(c)** $z_{0}=8$.
2. By using the complex method, we achieve to obtain simply periodic solutions $W_{s}(z)$ of the YSTF equation. The solutions $W_{s}(z)$ are in terms of the hyperbolic function solution. The solutions $W_{s}(z)$ in Figure 2 of the YSTF equation are represented the singular soliton solution for the parameters $k=1$, $l=1$, $r=1$, $\alpha=1$ and $y=0$ within the interval $-2\pi\leq\xi, \eta\leq2\pi$.

**Figure 2:**
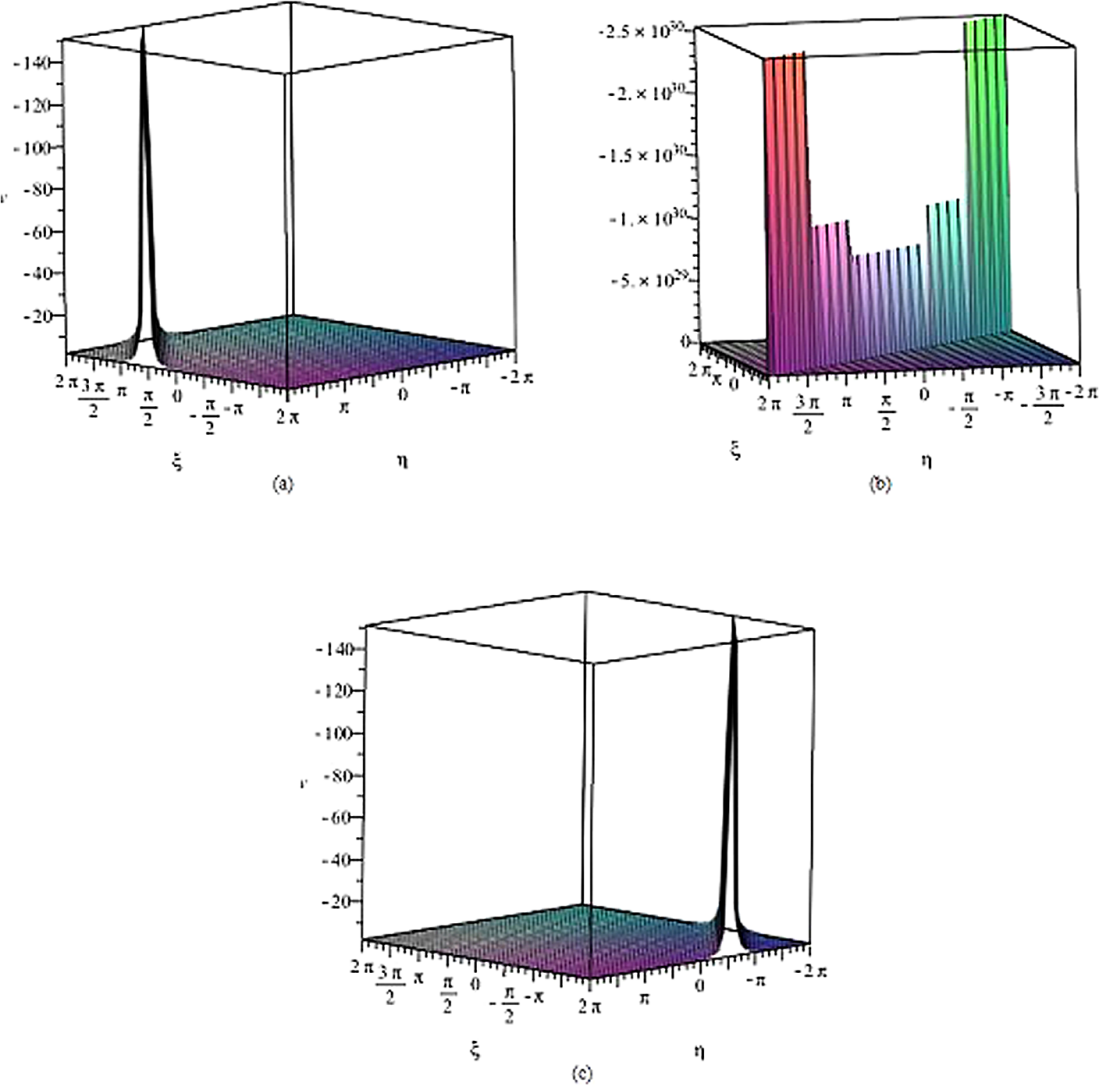
**The solution of the YSTF equation corresponding to** $\pmb{W_{s}(z)}$ **.** **(a)** $z_{0}=-8$, **(b)** $z_{0}=0$, **(c)** $z_{0}=8$.

## Conclusions

In this article, we utilize Lie group analysis to obtain symmetries and symmetry reduction for two higher-dimensional NLEEs. In this way, we can reduce the dimension of the NLEEs, which is relevant in the fields of mathematical physics and engineering. Five types of explicit function solutions are constructed by the $\exp(-\phi(z))$-expansion method and complex method. It demonstrates these methods are very efficient and powerful to seek the exact solutions of NLEEs. We can apply the idea of this study to other NLEEs.
